# Inferences on the Nature of a Cr(V) or Cr(IV) Species Formed by Reduction of Dichromate by a Bovine Liver Homogenate: NMR and Mass-Spectrometric Studies

**DOI:** 10.1155/S1565363303000220

**Published:** 2003

**Authors:** Elena Gaggelli, Nicola D'Amelio, Nicola Gaggelli, Gianni Valensin, Lucia Bovalini, Alessandro Paffetti, Lorenza Trabalzini

**Affiliations:** 1 Department of Chemistry University of Siena Via A.Moro Siena 53100 Italy; 2 Department of Molecular Biology University of Siena Via Fiorentina Siena 53100 Italy

## Abstract

A low-molecular weight chromium-containing fraction of the material resulting from dichromate reduction by bovine liver homogenate was investigated by NMR and ES-MS. The ES-MS spectrum showed a readily detectable peak at m/z = 786.1. The same molecular weight reasonably agreed with the relatively low diffusion coefficient measured by NMR-DOSY experiments on the main species observed in the ^1^H NMR spectrum. At least two downfield shifted and broad paramagnetic signals were apparent in the ^1^H NMR spectrum. Temperature dependence of chemical shift was exploited in order to estimate the diamagnetic shift of the signals in the diamagnetic region of the spectrum. 2D TOCSY, NOESY, COSY and ^1^H-^3^C HMQC spectra revealed the presence of aromatic protons (which were assigned as His residues), Gly and some other short chain amino-acids. Combinations of the molecular masses of such components together with acetate (which is present in the solution) and chromium atoms allowed a tentative proposal of a model for the compound.


### Inferences on the Nature of a Cr(V) or Cr(IV) Species Formed by Reduction of Dichromate by a Bovine Liver Homogenate: NMR and Mass-Spectrometric Studies

Elena Gaggelli, Nicola D'Amelio, Nicola Gaggelli, Gianni Valensin*

*Department of Chemistry, University of Siena, Via A.Moro, Siena 53100, Italy*

Lucia Bovalini, Alessandro Paffetti, Lorenza Trabalzini

*Department of Molecular Biology, University of Siena, Via Fiorentina, Siena 53100, Italy*

### ABSTRACT

A low-molecular weight chromium-containing fraction of the material resulting from dichromate reduction by bovine liver homogenate was investigated by NMR and ES-MS. The ES-MS spectrum showed a readily detectable peak at m/z = 786.1. The same molecular weight reasonably agreed with the relatively low diffusion coefficient measured by NMR-DOSY experiments on the main species observed in the $^1H$ NMR spectrum. At least two downfield shifted and broad paramagnetic signals were apparent in the $^1H$ NMR spectrum. Temperature dependence of chemical shift was exploited in order to estimate the diamagnetic shift of the signals in the diamagnetic region of the spectrum. 2D TOCSY, NOESY, COSY and $^1H$-$^{13}C$ HMQC spectra revealed the presence of aromatic protons (which were assigned as His residues), Gly and some other short chain amino-acids. Combinations of the molecular masses of such components together with acetate (which is present in the solution) and chromium atoms allowed a tentative proposal of a model for the compound.

### INTRODUCTION

Historically, chromium has been the first metal to be recognized as carcinogenic and mutagenic /1/. Beyond the risk associated with inhalation of $Cr^0$ particles, the adverse biological effects are ascribed to $Cr^{VI}$ compounds, since chromate or dichromate may readily enter cells through the sulfate channel /2/. According to the uptake-reduction model /3/, intracellular reduction of $Cr^{VI}$ activates the cascade of reactions leading to toxicity; whereas $Cr^{III}$ compounds are not able to cross the cytoplasmic membrane and may be therefore considered devoid of adverse effects. The beneficial effects of $Cr^{III}$ compounds and the eventual occurrence

* Corresponding Author: Phone: ++39-0577-234231; Fax: ++39-0577-234254; e-mail: valensin@unisi.it of $Cr^{III}$-containing natural macromolecules have been given great prominence in the bioinorganic chemistry literature /4-25/.

Within cells, $Cr^{VI}$ is reduced to lower valence states by several potential reductants, including cellular thiols, mainly cysteine and glutathione, GSH, NADPH and ascorbate /26, 27/, with formation of reactive oxygen species, thiyl and carbon-based radicals /28, 29/. In exposed animals, the ultimate steps of the metabolic pathway are characterized by $Cr^{III}$ insertion in the cell nucleus, where it cross-links DNA to proteins or GSH itself /30, 31/.

Reduction of chromate by GSH has been thoroughly investigated *in vitro* /26, 32-40/. It has emerged that, when reducing chromate, GSH first forms a thioester transient species and then a $Cr^{V}$ complex where two GSH molecules are able to cluster two metal ions /38/ with the eventual participation of aspartic acid as clustering agent /39/. The reported investigations have not succeeded so far in ascertaining whether this complex is relevant for detoxification or if it is just an intermediate step in the reductive pathway to mutagenic $Cr^{III}$. In the present report, we have investigated the reduction of $Cr^{VI}$ by the bovine liver homogenate, where GSH is expected to occur at high concentrations and it is known to actively participate in cell protection.

### MATERIALS AND METHODS

All chemicals were obtained from Sigma Chemical Co. and used without further purification.

The bovine liver (*ca.* 1 Kg) was suspended in 1 liter of a 3.4 mM solution of $K_2Cr_2O_7$ containing protease inhibitors and homogenized through mechanical high velocity agitation for about 60 s. The homogenate was centrifuged at about 11000*g* for 10 min at 4°C. All subsequent procedures were performed at 4°C. An equal volume of ethanol was added to the supernatant and the resulting slurry was agitated for 12 h and then centrifuged at 11000*g* for 10 min. The new supernatant was mixed to more ethanol so as to reach a 90% solution and kept under agitation for 2 days. A light brown precipitate was obtained and separated through centrifugation at 4200*g* for 5 min. The precipitate was freeze dried until a golden brown solid was obtained, extracted with a minimum volume of water, shortly centrifuged and filtered through glass wool. The clear greenish brown filtrate was loaded on a Whatman column (ca. 2.5 x 80 cm) of DEAE-cellulose equilibrated with an ammonium acetate 0.2 M buffer solution, pH 7.2. The column was washed with 1 liter of the buffer and eluted with a 1-L linear gradient from 0.2 to 2.0 M ammonium acetate, pH 7.2. The chromium concentration in this and subsequent columns was monitored using the absorbance at 260 nm. Fractions containing the major Cr concentrations were pooled and concentrated by ultrafiltration (Amicon 8010 using YC05 membrane), diluted with an equal volume of water and applied again to an identical DEAE column, washed and eluted as previously described. Chromium rich fractions were again pooled and concentrated by ultrafiltration. The gray green solution was applied to a Sephadex G-25 column (ca. 2.5 × 80 cm) and eluted with ammonium acetate 0.05 M, pH 6.5. Chromium containing fractions were collected and concentrated by ultrafiltration (Amicon 8010 and/or 8400 with YC05 membrane) to less than 10 mL. The deep gray-green solution was applied to a Sephadex G-15 column (ca. 6.5 × 60 cm) and eluted with ammonium acetate 0.05

M, pH 6.5. Gray-green fractions were again collected, concentrated and reapplied to the same G-15 column. The resulting solution was concentrated by ultrafiltration, lyophilized and stored at - 20°C.

ES-MS spectra were recorded with a Perkin-Elmer Sciex triple quadrupole liquid chromatography/mass spectrometry/mass spectrometry (LC/MS/MS) with a water/formic acid (equilibrated at pH 7.4) mobile phase.

$^1$H-NMR spectra were obtained at 14.1 T with a Bruker Avance 600 Spectrometer operating at controlled temperature (± 0.2 K). Chemical shifts were referenced to external TMS (tetramethylsilane). Water suppression was achieved either with presaturation or with excitation sculpting /41/ using a selective square pulse on water 2ms long. COSY, TOCSY, NOESY and HMQC spectra were obtained by using standard pulse sequences. TOCSY experiments were acquired with a total spin-locking time of 75 ms using a MLEV-17 mixing sequence. 2D-NOESY was acquired with a mixing time of 150 ms.

DOSY spectra were acquired at 298 K using a 5 mm triple resonance probe with gradients along x,y,z directions. A PFG longitudinal eddy-current delay (LED) pulse sequence with bipolar gradients incorporating spoil gradients during both longitudinal storage periods was used /42,43,44/. Diffusion coefficients were measured by incrementing the gradient strength (with an initial value of 0.86 G $cm^{-1}$ and a step size of 2.65 G $cm^{-1}$ for 2 ms), while the separations (250 ms) of the field gradients and the total echo time were kept constant. A series of 16 spectra with 128 scans was recorded in 2D mode for each measurement, with a recycle time of 5 s between scans. The strength of the $B_0$ field gradient was calibrated by measuring the self-diffusion coefficient of the residual HDO signal in a 100% $D_2O$ sample at 298 K /45/. A diffusion coefficient D of $1.90 \times 10^{-9}$ $m^2$ $s^{-1}$ /46/ was used for back calculation of the gradient strength by fitting peak volumes (without manipulating the window function) to a sigmoid curve (eq. [2] in the text). The same measurement was used to determine the hydrodynamic radius of water at 0.101 nm using a viscosity for $D_2O$ at 298 K of $1.132 \times 10^{-3}$ Kg $m^{-1}$ $s^{-1}$ /47/ (eq. [1] in the text). A diffusion experiment was performed on a sample of TSP in $D_2O$ at 298 K in order to evaluate its hydrodynamic radius r by the ratio $r_{TSP} = r_{water}D_{water}/D_{TSP}$ /48/ at 0.351 nm. Such value was then used to measure the viscosity of the solution containing the material extracted from bovine liver by measuring the diffusion of TSP (eq. [1] in the text).

### RESULTS AND DISCUSSION

The material resulting from dichromate reduction within the bovine liver homogenate was previously shown to display the following features /39/:

(i) UV-vis spectra are very similar to those obtained when reducing chromate with GSH at pH 7.4 and exclude the occurrence of any $Cr^{III}$ compound, thus suggesting that the +V or +IV states have been stabilized;

(ii) EPR spectra are again very similar (the same g value (1.93), a similar hyperfine structure consistent with coupling with two almost equivalent nitrogens and only a slightly different linewidth) to those obtained from the GSH/chromate system at pH 7.4. It may thus be suggested that a similar cluster of two $Cr^V$ ions is very likely to occur in the liver homogenate.

(iii) The 800 MHz $^1$H-NMR spectrum of the chromium-containing liver homogenate dissolved in $H_2O$ (with 10% of $D_2O$) at pH 7.4 shows at least two large upfield shifted signals (in the range –20 ↔ -40 ppm) demonstrating the presence of some paramagnetic species, which, again, is not consistent with the presence of $Cr^{III}$ compounds.

The ES-MS spectrum of the chromium-containing material showed a readily detectable peak at m/z = 786.4 (Figure 1), the two signals at 808.8 and 824.4 being accounted for by the assumption of one sodium or one potassium atom respectively. Again, as shown by the theoretical fingerprint of the peak, occurrence of a homonuclear chromium dimer is suggested in very close agreement with what was observed with GSH alone. As will be later discussed, these findings suggest the presence of a high molecular weight compound to which the upfield shifted signals appearing in the NMR spectrum (data not shown) may belong.

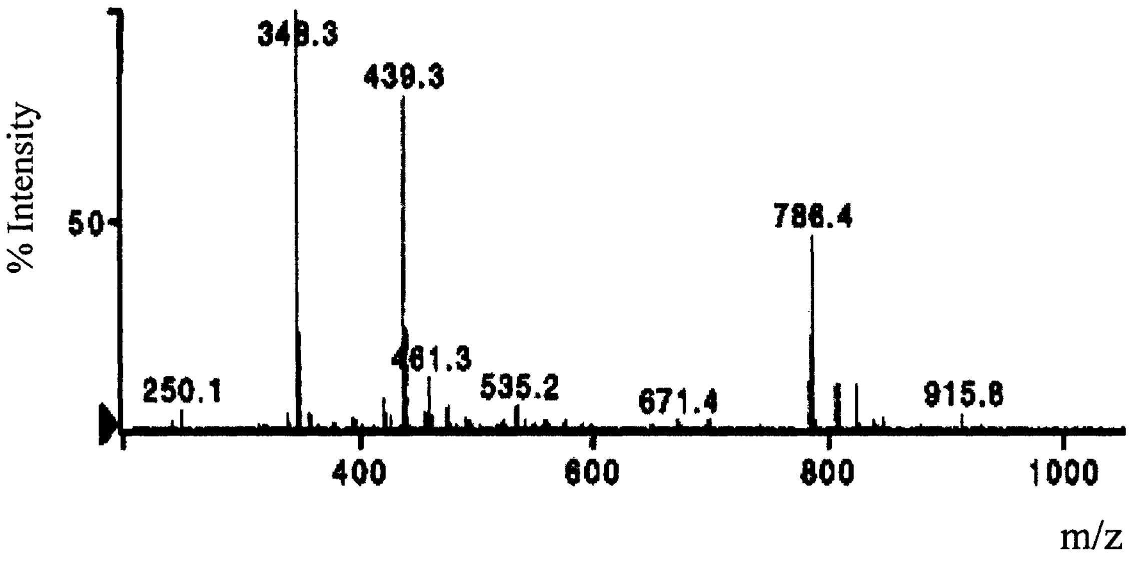

**Fig. 1:** Region of ES-MS spectrum of the material extracted from liver.

The diamagnetic region (0 – 10 ppm) of the 600 MHz $^1$H-NMR spectrum is shown in Figure 2. The signals can be grouped into three sets:

a) High intensity signals at 1.98 ppm (assigned to the methyl group of acetate) and 3.5 – 3.8 ppm besides the strong water signal that had to be suppressed; b) medium intensity signals (later considered) in some cases very broad; c) very low intensity signals flanking all the signals in b) as they belonged to a similar isomeric species or a different complex with a slightly different coordination mode; one peak, flanking the acetate resonance at 1.98 ppm, could originate from a second form of the acetate or belong to set b.

Due to the unknown nature of species occurring in the chromium containing fraction of the liver homogenate, we decided first to assess whether the observed signals belong to the same species. A DOSY experiment was therefore performed on the material as shown in Figure 3 /49, 50/. Transport properties of molecules and ions are in fact connected with structural properties because diffusion coefficients (D) depend

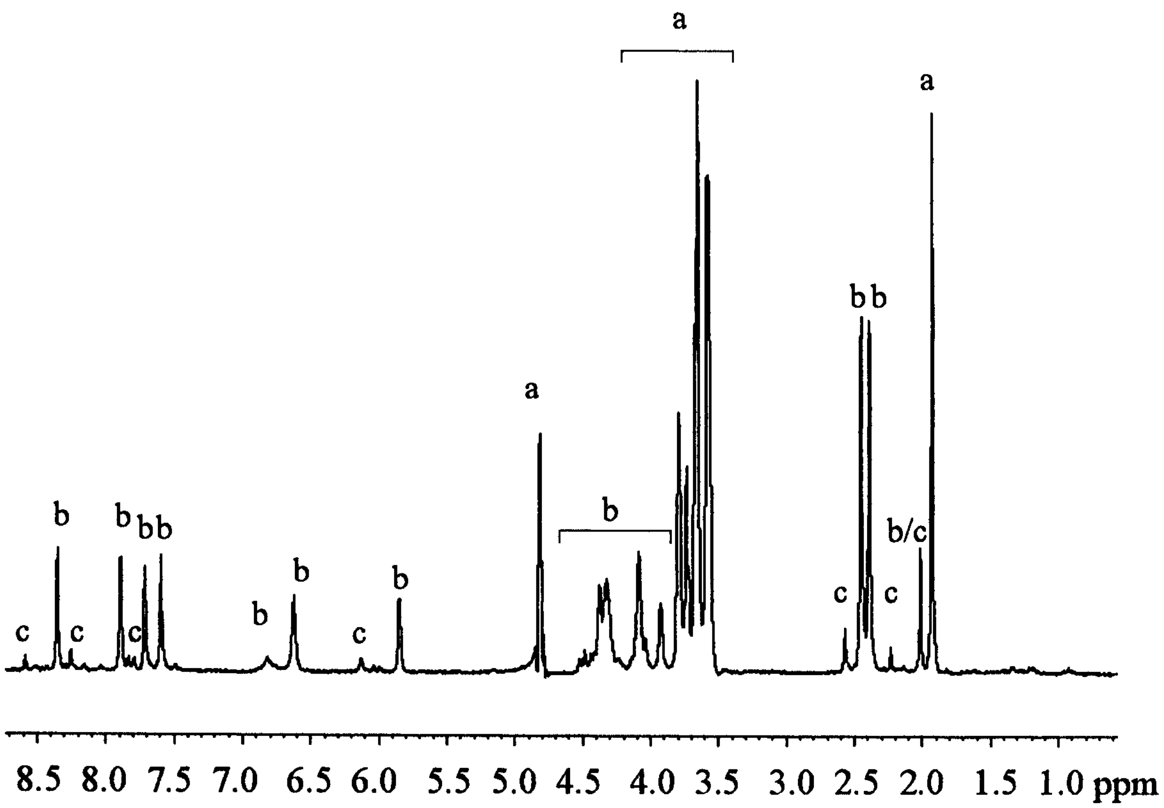

**Fig. 2:** $^1$H 600 MHz spectrum of the material extracted from liver in $H_2O$ (containing 10% $D_2O$) at pH 7.35, T=298 K.

on friction factors ($f_T$):

$$D = \frac{k_B T}{f_T} \qquad f_T = 6\pi\eta r_H \tag{1}$$

where $k_B$ is the Boltzmann constant, $\eta$ is the viscosity of the medium and $r_H$ is the hydrodynamic radius of the molecule, here assumed of spherical shape /51/. The result is a diffusion-ordered 2D map where signals belonging to molecules having the same D value are all found on the same horizontal line.

The map reported in Figure 3 shows that the line corresponding to the slowest diffusion coefficient (3 x $10^{-10}$ $m^2$/ s) contains most of the medium size signals of the 1D spectrum suggesting that they all belong to the same molecule. Other apparent peaks belong to water (4.7 ppm) and to acetate (1.9 ppm) which is used in the extraction procedure. The other few peaks belong to signals too weak to be considered. In order to better determine the value of the diffusion constant the intensity of the signals at D=$3\times10^{-10}$ $m^2$/ s was plotted as a function of the gradient strength G and regression analysis was performed with the equation /52/:

$$I = I_0 \exp[-Dq^2(\Delta - \delta/3 - \tau/2)] \tag{2}$$

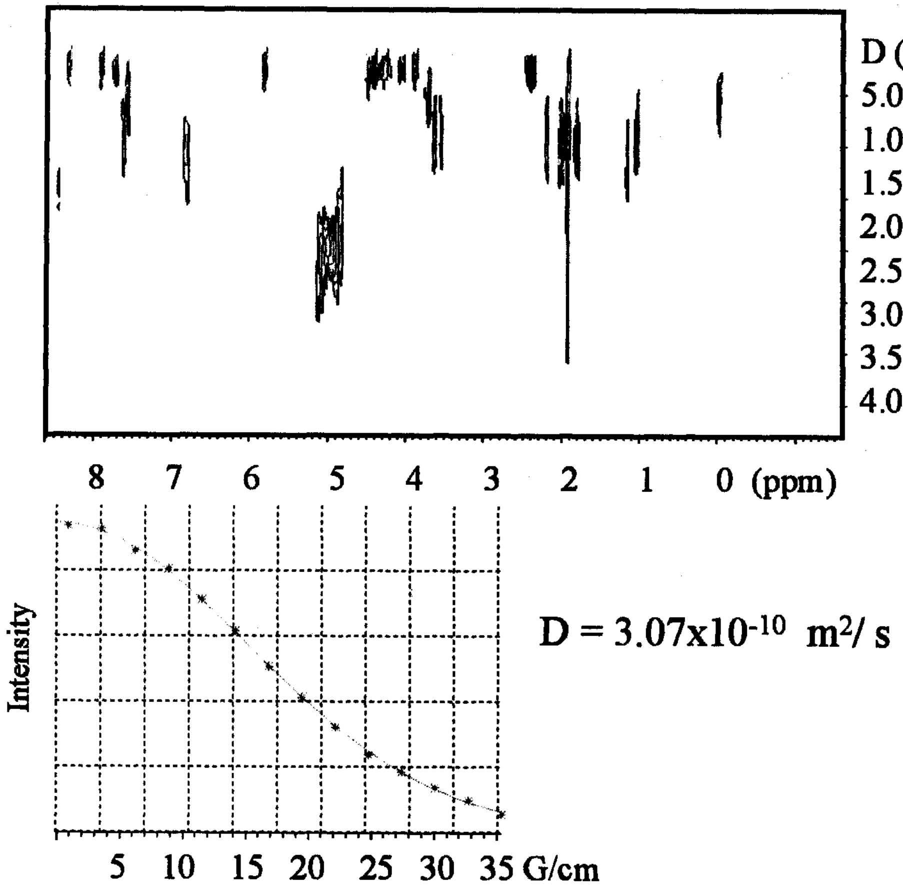

**Fig. 3:** (top) DOSY spectrum at 600 MHz of the material extracted from liver in $H_2O$ (containing 10% $D_2O$) at pH 7.35, T=298 K and (bottom) regression analysis of the curve obtained by plotting the intensity of one peak belonging to the species with lower diffusion as a function of the gradient strength used in the DOSY experiment.

where I and $I_0$ are the intensities of the signal for each gradient strength used in the experiment and for 2% gradient strength, $\Delta$ and $\delta$ are the big and the little delta of the BPP-LED experiment which represent respectively the interval between the two bipolar composite gradients and the duration of each gradient within each bipolar composite gradient, $\tau$ is the gradient pulse separation and $q = 2\pi\gamma G\delta$. An average value of $3.15\times10^{-10}$ $m^2/s$ was obtained from all the peaks belonging to the upper line in the DOSY experiment. In order to roughly estimate the molecular weight of the species observed, the apparent molecular weight M was calculated as /48,53/:

$$M = \left(\frac{k_B T}{6\eta\pi F D}\right)^3 \left[\frac{4\pi N_A}{3(\upsilon_2 + \delta_1 \upsilon_1)}\right] \qquad (3)$$

where $k_B$ is the Boltzmann constant, T is the absolute temperature, $\eta$ is the viscosity of the solution, $N_A$ is Avogadro's number, $v_2$ and $v_1$ are the partial specific volumes of the molecule and solvent water, respectively, and $\delta_1$ is the fractional amount of water bound to the molecule (hydration number). F is the shape factor, or Perrin factor, which is defined to be the ratio of the friction coefficient of the molecule to that of a hard sphere with equivalent mass and partial specific volume. The viscosity of the solution ($9.77 \times 10^{-4}$ Kg $m^{-1}$ $s^{-1}$) was determined by the diffusion coefficients of the internal reference TSP as described in the experimental section. We considered an axial ratio of 1 (F=1), $0.73 \times 10^{-6}$ $m^3g^{-1}$ and $1 \times 10^{-6}$ $m^3g^{-1}$ were used for the partial specific volumes $v_2$ and $v_1$ of the material and the solvent /54/, and a value of 0.4 was used for the hydration number (hydration numbers in the range 0.3–0.4 gram of water per gram of protein are common for most proteins) /48/. The apparent molecular value was found at 796.5. Such value provides an upper limit, since eventual relatively large errors on the hydration number and/or the shape factor yield lower molecular weights. It follows that what is inferred from diffusion measurements is anyway in fairly good agreement with the molecular weight observed in the ES-MS spectrum, thus indicating that the set of peaks in the DOSY spectrum and the ES-MS signal at m/z = 786.4 refer to the same species, which, most likely, brings a unit charge.

In order to assess the amount of paramagnetic contribution for the protons in the diamagnetic region of the spectrum the temperature dependence of chemical shifts was studied (Figure 4). Diamagnetic chemical shifts of aliphatic protons should in fact have poor temperature dependence while both contact and pseudo-

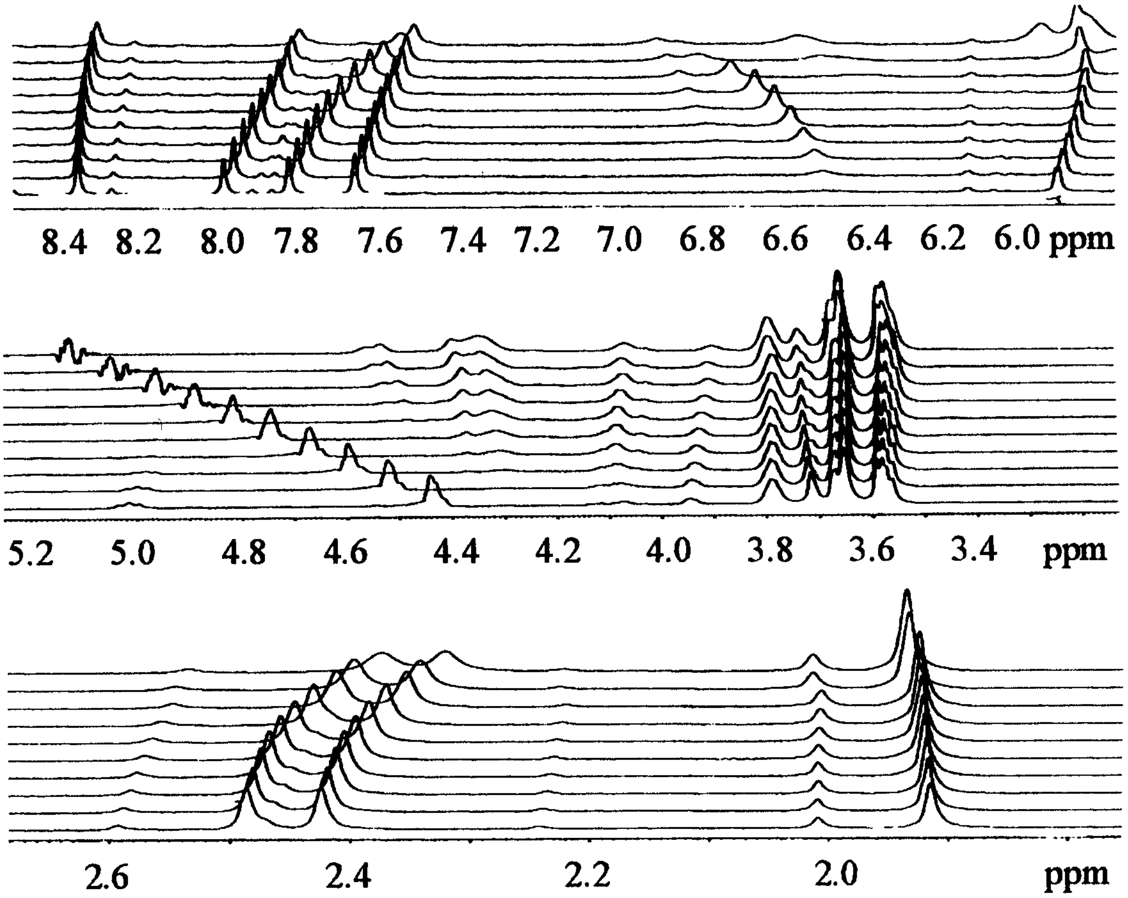

**Fig. 4:** Temperature dependence from 280 (top) to 325 K (bottom) in steps of 5 K of 600 MHz $^1H$ spectra of the material extracted from liver in $H_2O$ (containing 10% $D_2O$) at pH 7.35.

contact chemical shifts are inversely proportional to the temperature. In particular, when the zero field splitting (ZFS) is negligible (as in the presence of only one unpaired electron) and in case of axial symmetry of the g tensor the following equations hold /55/:

$$\delta^p = \delta^c + \delta^{pc} = \frac{A}{\hbar}\frac{g_{iso}\mu_B S(S+1)}{3\gamma_I k_B T} + \frac{A^{pc}}{\hbar}\frac{g_{iso}\mu_B S(S+1)}{3\gamma_I k_B T} = \frac{const}{T}$$

$$A^{pc} = \frac{\mu_0}{12\pi}\hbar\gamma_I \bar{g}\mu_B\left(\frac{g_{\parallel}^2}{\bar{g}^2} - \frac{g_{\perp}^2}{\bar{g}^2}\right)\frac{1}{r^3}(3\cos^2\theta - 1) \qquad (4)$$

where A and $A^{pc}$ are the electron-nucleus contact and pseudocontact coupling constants respectively, $\mu_o$ is the permeability of vacuum, S is the spin quantum number of the paramagnetic species, $\mu_B$ is the electron Bohr magneton, $g_{iso}$, $g_{\parallel}$ and $g_{\perp}$ are the isotropic parallel and perpendicular electron g factors, $\gamma_I$ is the proton magnetogyric ratio, θ is the angle between the metal-nucleus vector and the molecular z axis and p, c and pc superscripts stand for paramagnetic, contact and pseudo-contact.

Eqs. (4) show that the paramagnetic contribution to the chemical shifts should follow an hyperbolic law as a function of temperature. Figure 5 shows the temperature dependencies of the chemical shifts of some representative proton signals belonging to set b). It is clearly seen that some protons at 320 K are very near the plateau, meaning that the value of the chemical shift is practically not affected by the paramagnetic contribution. In other cases fitting the curves with an hyperbolic curve has made it possible to measure the limiting deviation from the chemical shift values in the absence of paramagnetic contribution. Results are reported in Table 1. The extrapolated diamagnetic shifts can be used to assign NMR peaks to some spin

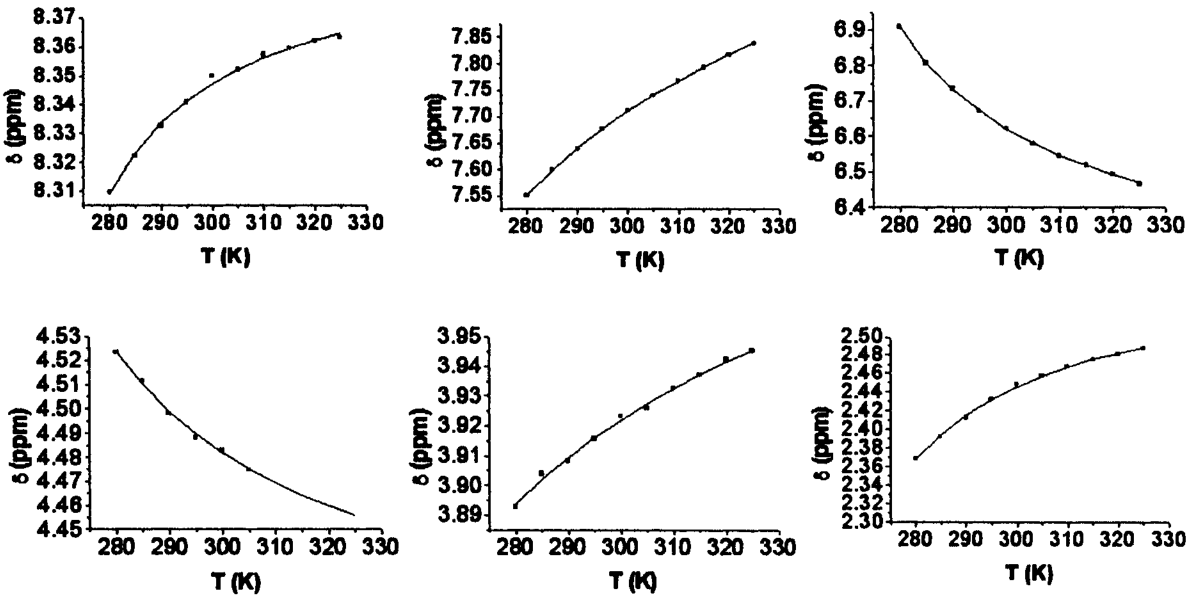

**Fig. 5:** Temperature dependence of chemical shifts of some representative peaks with shorter diffusion constant of the material extracted from liver in $H_2O$ (containing 10% $D_2O$) at pH 7.35.

**Table 1**

$^1$H-NMR chemical shifts measured at 300 K in the diamagnetic region of the 600 MHz $^1$H-NMR spectrum of the chromium-containing material obtained upon reduction of dichromate by the bovine liver homogenate. The third and fourth columns report the chemical shifts and the deviations measured at the plateau of the temperature dependent curves. Diffusion coefficients derived from each peak are reported in the last column. Errors on $^1$H chemical shifts can be estimated as ± 0.05 ppm while errors on diffusion coefficients were estimated around 3 %.

| Chemical shift at 300 K (ppm) | Temptative assignment | Limiting value (diamagnetic shift, ppm) | Deviation (ppm) | Diffusion coefficient $(m^2/s) \times 10^{-10}$ |
|---|---|---|---|---|
| 8.35 | Amide proton | 8.39 | -0.04 | 3.12 |
| 7.89 | Amide proton | 8.34 | -0.45 | 3.15 |
| 7.71 | $H_\delta$ His | 8.37 | -0.66 | 3.25 |
| 7.59 | $H_\delta$ His | 7.95 | -0.36 | - |
| 6.88 | $H_\varepsilon$ His | - | - | - |
| 6.82 | $H_\varepsilon$ His | - | - | - |
| 6.62 | - | 6.14 | 0.48 | - |
| 4.48 | $H_\alpha$ | 4.39 | 0.09 | 3.14 |
| 4.37 | $H_\alpha$ Gly | 4.34 | 0.03 | 3.10 |
| 3.92 | $H_\alpha$ Gly | 4.05 | -0.13 | 3.09 |
| 2.45 | $H_\beta$ His | 2.58 | -0.13 | 3.16 |
| 2.39 | $H_\beta$ His | 2.53 | -0.14 | 3.16 |
| 1.91 | acetate | - | - | 9.98 |
| 0.00 | TSP | - | - | 6.37 |

system with the aid of 2D spectroscopy, although the interaction with the metal ion charge may, by itself, lead to sizeable changes in chemical shift. It must however be kept in mind that the largest variations are most probably experienced by nuclei in the immediate surrounding of the paramagnetic ion and that these are hardly observed due to fast transverse relaxation caused by the coupling with the electron spin magnetic moment.

The temperature dependence of chemical shifts provides further information based on the sign of the deviations which is dependent on the angle $\theta$ in eqs. (4) and can help in connecting spin systems as near nuclei should experience similar shifts.

Analysis of data shows that, on all peaks, the paramagnetic shift is restricted within 0.5 ppm making the regions of the different types of protons basically unchanged. The presence of consistent broadening in the alpha and aromatic proton regions of peptides suggests that, besides binding to backbone or side-chain aliphatic donors, coordination to atoms on aromatic side chains as Tyr, His or Trp is likely to occur. In particular TOCSY, NOESY and HMQC spectra (Figure 6) are consistent with the presence of at least two different His residues. Figure 6a shows the TOCSY spin systems of the aromatic rings connecting $H_\varepsilon$ and $H_\delta$; in addition a TOCSY cross-peak is present between the narrower of each pair of signals and the $\beta$ protons.

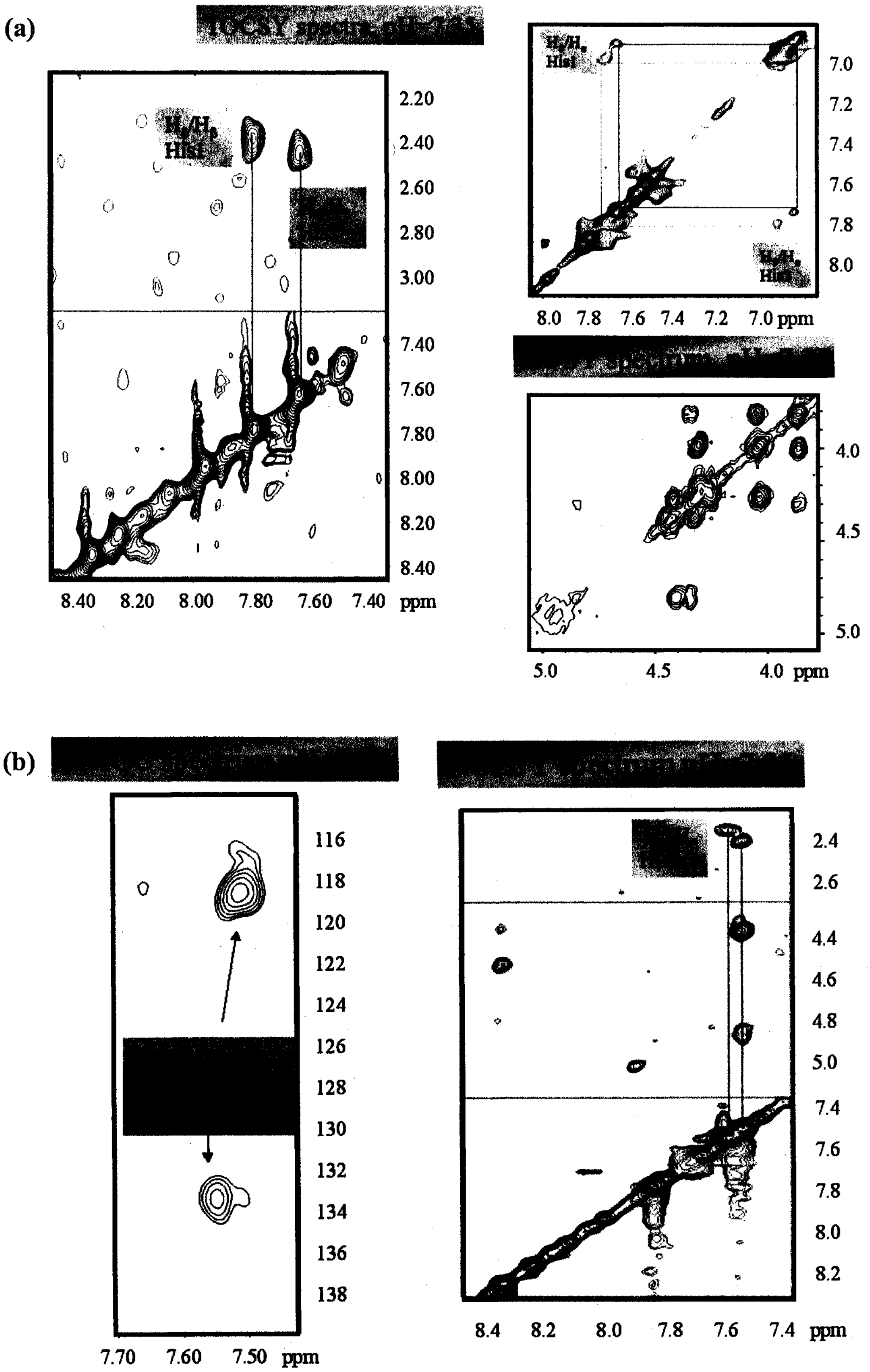

**Fig. 6:** 2D 600 MHz spectra of the material extracted from liver in $H_2O$ (containing 10% $D_2O$) at pH 7.35. (a) TOCSY and COSY regions (T=298 K), (b) HMQC and NOESY regions (T=278 K).

The $H_\delta$-$H_\beta$ TOCSY cross–peaks can only be found in His or Trp spin systems (not for Phe and Tyr), but occurrence of Trp can be discarded as no other aromatics protons are present. The broadening of $H_\varepsilon$ protons together with the stronger deviation (Table 1) observed for $H_\delta$ with respect to $H_\beta$ indicate the aromatic ring as a possible chromium-bonding moiety. The absence of $H_\alpha$ and HN signals can be originated by broadening and further bonding to amide proton cannot be excluded. HMQC spectrum (Figure 6b) reveals that among the four protons within 7.0 and 8.5 ppm only those at 7.71 and 7.59 ppm are directly linked to carbon atoms, thus suggesting that peaks at 8.35 and 7.89 ppm belong to two amide protons. COSY and TOCSY spectra do not reveal any connectivity to these two signals; only two NOESY cross-peaks connect the first signal with the one at 4.48 ppm (strong) and the one at 4.37 ppm (weak) as reported in Figure 6b. The latter is connected in the NOESY spectrum also with one $H_\delta$ of His, and most importantly has a COSY cross-peak with the signal at 4.48 ppm suggesting the presence of a Gly. As indicated in Table 1, all detectable protons (with the exception of the signals assigned to His and amide protons) have limiting values of diamagnetic shifts between 4.0 and 4.4 ppm. If amino-acids are exclusively considered, this ppm range competes to $\alpha$ protons only. This situation can be due to the presence of Gly residues or to short side-chain peptides such as Asp, Asn, Ser or Cys, where $\beta$ protons can be washed out by line broadening, leaving only $\alpha$ protons detectable. However, as shown in Figure 6a, most of the signals between 4.0 and 4.4 ppm display COSY connectivities among themselves, supporting the hypothesis of the presence of Gly residues.

In order to get some more insight into the composition of the material, we tried to sum up all possible combinations of the masses of the possible amino acid components (Gly, His, Ser, Asn, Asp, Cys) which, together with acetate and two Cr atoms, could result in the molecular mass observed in the ES-MS experiment. Acetate was considered because is included in the preparation protocol and it is well known as an efficient clustering ligand for chromium /56/.

It was found that a combination of two His, two Gly, two Cys, two Cr atoms and one acetate molecule results in a molecular mass of 786.0397 (average molecular mass 786.685, molecular formula $C_{24}H_{30}N_{10}O_{10}S_2Cr_2$) provided four water molecules (for the formation of four peptide bonds) and nine protons are removed. This is possible by assuming acetate bridging two Cr atoms coordinated by two $N_\varepsilon$ of the His rings ($H_\varepsilon$ is actually very broad), two sulfurs of the Cys residues (which would explain why $H_\beta$ are missing in the diamagnetic region of the NMR spectrum and could identify the broad upfield shifted peaks), and five ionized amino or amide nitrogens. In addition, since the total charge must be mono-positive, chromium is forced to assume a +5 oxidation state thus justifying the similarities with the $Cr^V$ dimer formed with GSH.

### INFERENCES AND CONCLUSIONS

The composition suggested by the coupled analysis of ES-MS and NMR data may lead to a hint of the presence of two small peptides binding the two bridged chromium atoms in a slightly asymmetric way. We suggest here that such small peptides might correspond to the Cys-His-Gly sequence found in domain III of bovine serum albumin (BSA, 245-247 fragment), which is particularly abundant in liver. The complex might therefore originate by degradation of the protein caused by chromate. As a matter of fact, it has recently been found that a copper(II) complex acts as an artificial metalloprotease towards BSA and that copper is subsequently found ligated to the nitrogen of histidine in domain III in several proteolytic fragments /57/. The picture emerging from these considerations is consistent with the presence of a $Cr^V$ dimer ligating two Cys-His-Gly peptides and one bridging acetate ion. Differences in the chemical shift of the two histidine residues, and the fact that an odd number of protons must be subtracted for obtaining the experimental ES-MS spectrum, suggests that the coordination mode is asymmetric. Furthermore, since bridging of acetate is known to yield Cr atoms very close to each other, the broader EPR signal /39/ of the Cr-containing material extracted from bovine liver homogenate in respect of the $Cr^V$ dimers obtained with GSH or GSH/Asp may be accounted for as well. As a consequence, a hypothetic tentative structure of the complex was built as shown in Figure 7.

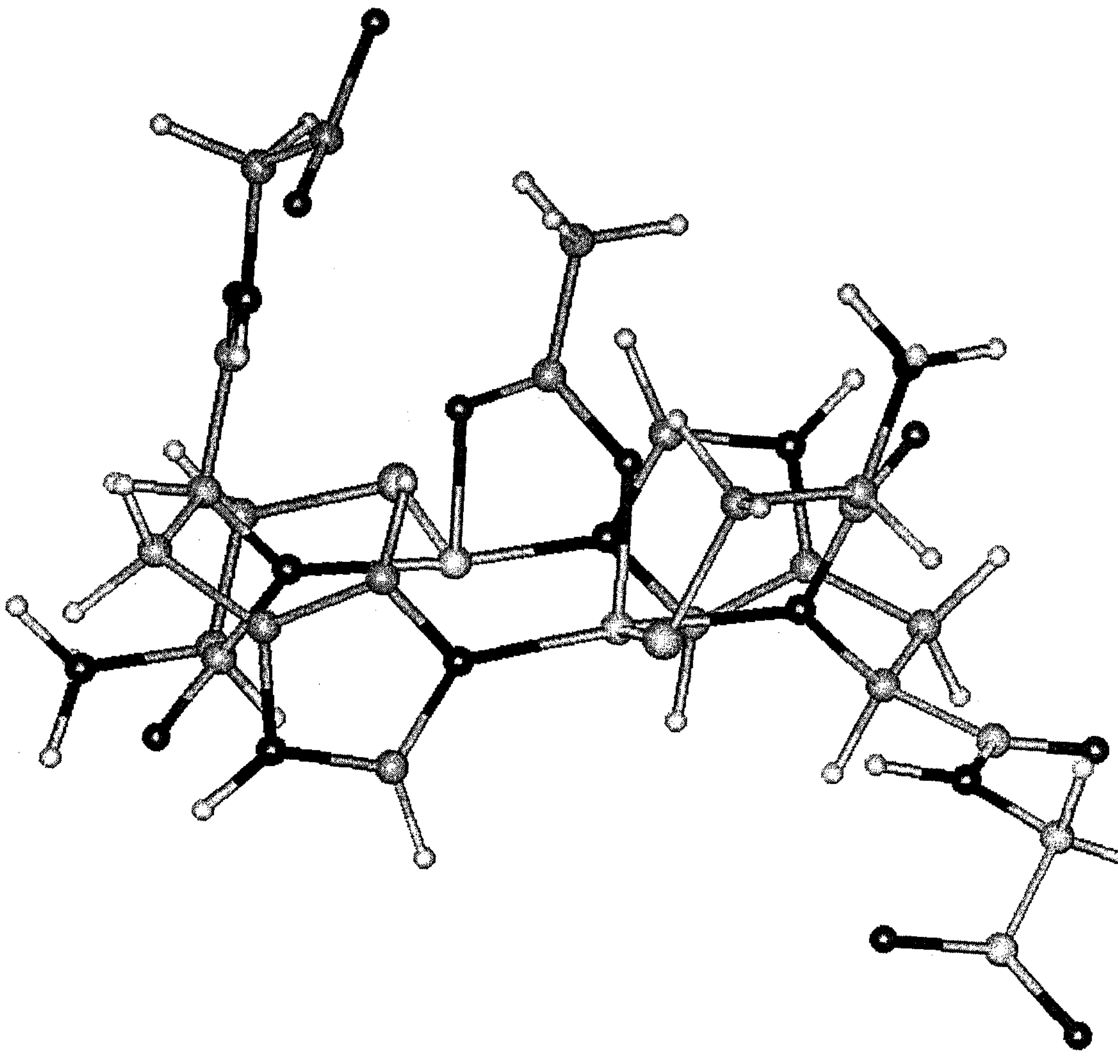

**Fig. 7:** Hypothetic tentative model of a chromium(V) dimer ligated by two Cys-His-Gly peptides and bridged by an acetate ion. Water molecules are likely to complete the coordination sphere of the two metal ions. The model was created with HYPERCHEM software package /58/.

However, it is worth remarking that the suggested species is not the only one occurring in solution, since the presence of the aforementioned small-intensity satellite peaks implies the occurrence of at least one isomeric form of the complex.

### REFERENCES

1. IARC, *IARC Monographs on the Evaluation of the Carcinogenic Risk of Chemicals to Humans: Chromium, Nickel and Welding.* IARC, Lyon, 1-214 (1990).
2. H.J. Wiegand, H. Ottenwälder and H.M. Bolt, *Sci.Total Environ.*, **71**, 309-315 (1988).
3. P.H. Connett and K.E. Wetterhahn, *Struct.Bond. (Berlin)*, **54**, 93-124 (1983).
4. E.W. Toepfer, W. Mertz, M.M. Polansky, E.E. Roginski and W.R. Wolf, *J.Agric. Food Chem.*, **25**, 162-166 (1977).
5. O. Wada, G.Y. Wu, A. Yamamoto, S. Manabe and T. Ono, *Environ.Res.*, **32**, 228-239 (1983).
6. R.A. Anderson, M.M. Polansky, N.A. Bryden, E.E. Roginski, W. Mertz and W. Glinsmann, *Metabolism*, **32**, 894-899 (1983).
7. A. Yamamoto, O. Wada and T. Ono, *J.Inorg.Biochem.*, **22**, 91-102 (1984).
8. A. Yamamoto, O. Wada and T. Ono, *Eur.J.Biochem.*, **165**, 627-631 (1987).
9. A. Yamamoto, O. Wada and H. Suzuki, *J.Nutr.*, **118**, 39-45 (1988).
10. A. Yamamoto, O. Wada and S. Manabe, *Biochem.Biophys.Res.Commun.*, **163**, 189-193 (1989).
11. D.M. Stearns and W.H. Armstrong, *Inorg.Chem.*, **31**, 5178-5184 (1992).
12. G.W. Evans and T.D. Bowman, *J.Inorg.Biochem.*, **46**, 243-250 (1992).
13. M.C. Davis, K.H. Sumrall and J.B. Vincent, *Biochemistry*, **35**, 12963-12969 (1996).
14. M.C. Davis and J.B. Vincent, *Arch.Biochem.Biophys*, **339**, 335-343 (1997).
15. R.A. Anderson, *J.Am.Coll.Nutr.*, **16**, 404-410 (1997).
16. R.A. Anderson, N.A. Bryden and M.M. Polansky, *J.Am.Coll.Nutr.*, **16**, 273-279 (1997).
17. M.C. Davis and J.B. Vincent, *J.Biol.Inorg.Chem.*, **2**, 675-679 (1997).
18. M.C. Davis and J.B. Vincent, *Biochemistry*, **36**, 4382-4385 (1997).
19. M.C. Davis, A.C. Royer and J.B. Vincent, *Inorg.Chem.*, **36**, 5316-5320 (1997).
20. K.H. Sumrall and J.B. Vincent, *Polyhedron*, **16**, 4171-4177 (1997).
21. J.K. Speetjens, R.A.Collins, J.B. Vincent and S.A. Woski, *Chem.Res.Toxicol.*, **12**, 483-487 (1999).
22. J.B. Vincent, *J.Am.Coll.Nutr.*, **18**, 6-12 (1999).
23. J.B. Vincent, *J.Nutr.*, **130**, 715-718 (2000).
24. J.B. Vincent, *Acc.Chem.Res.*, **33**, 503-510 (2000).
25. L. Jacquamet, Y. Sun, J. Hatfield, W. Gu, S.P. Cramer, M.W. Crowder, G.A. Lorigan, J.B. Vincent and J.-M. Latour, *J.Am.Chem.Soc.*, **125**, 774-780 (2003).
26. P.H. Connett and K.E. Wetterhahn, *J.Am.Chem.Soc.*, **107**, 4283-4288 (1985).
27. A. Zhitkovich, Y.Song, G. Quievryn and V. Voitkun, *Biochemistry*, **40**, 549-560 (2001).
28. P.C. Kaltreider, C.A. Pesce, M.A. Ihnat, J.P. Lariviere and J.W. Hamilton, *Mol. Carcinogenesis*, **25**, 219-229 (1999).

29. S. Liu, M. Medvedovic and K. Dixon, *Environ.Mol. Mutagenesis,* **33**, 313-319 (1999).
30. C.A. Miller, M.D. Cohen and M. Costa, *Carcinogenesis,* **12**, 269-276 (1991).
31. V. Voitkun, A. Zhitkovich and M. Costa, *Nucleic Acid Res.,* **26**, 2024-2030 (1998).
32. D.M. Goodgame and A.M.. Joy, *J.Inorg.Biochem.,* **26**, 219-224 (1986).
33. S. Kitagawa, H. Seki, F. Kametani and H. Sakurai, *Inorg.Chim.Acta,* **152**, 251-255 (1988).
34. P. O'Brien, J. Pratt, F.J. Swanson, P. Thornton and G. Wang, *Inorg.Chlm Acta,* **169**, 265-269 (1990).
35. S.L. Brauer and K.E. Wetterhahn, *J.Am.Chem.Soc.,* **113**, 3001-3007 (1991).
36. R.N. Bose, S. Moghaddas and E. Gelerinter, *Inorg.Chem,* **31**, 1987-1994 (1992).
37. S. Moghaddas, E. Gelerinter and R.N. Bose, *J.Inorg.Biochem,* **57**, 135-146 (1995).
38. E. Gaggelli, F. Berti, N. Gaggelli, A. Maccotta and G. Valensin, *J.Am.Chem.Soc.,* **123**, 8858-8859 (2001).
39. E. Gaggelli, F. Berti, N. D'Amelio, N. Gaggelli, G. Valensin, L. Bovalini, A. Paffetti and L. Trabalzini, *Environ.Health Persp.,* **110** (Suppl. 5), 733-738 (2002).
40. A. Levina, L. Zhang and P.A. Lay, *Inorg.Chem.,* **42**, 767-784 (2003).
41/ T. L Hwang. and A. J. Shaka,. *J. Magn. Reson. Series A*, **112**, 275-279 (1995).
42. D. Wu, A. Chen and C.S. Johnson Jr., *J. Magn. Reson. A*, **115**, 260-264 (1995).
43. S. J. Gibbs and C. S..Johnson Jr., *J. Magn. Reson.*, **93**, 395–402 (1991).
44. A. J. Dingley, J. P. Mackay, B. E. Chapman, M. B. Morris, P. W. Kuchel, B. D. Hambly and G. F. King *J. Biomol. NMR*, **6**, 321–328 (1995).
45. P.T. Callaghan, M. A. L. Gros and D. N. Pinder *J. Chem.Phys.*, **79**, 6372–6381 (1983).
46. L. G. Longsworth, *J. Phys. Chem.*, **64**, 1914–1917 (1960).
47. R. C. Weast *et al.*, *Handbook of Chemistry and Physics. A Ready-Reference Book of Chemical and Physical Data, 52nd edition,* The Chemical Rubber Company, Cleveland, Ohio (1971).
48. S. Yao, G. J. Howlett and R. S. Norton, Peptide self-association in aqueous trifluoroethanol monitored by pulsed field gradient NMR diffusion measurements, *Journal of Biomolecular NMR,* **16**, 109–119, (2000).
49. K.F. Morris and C.S. Johnson Jr., *J.Am.Chem.Soc.,* **114**, 3139- (1992).
50. C.S. Johnson Jr., *Progr.NMR Spectr.,* **34**, 203-256 (1999).
51. H.J.V. Tyrrell and K.R. Harris, *Diffusion in Liquids: A Theoretical and Experimental Study, Butterworths*, London (1984).
52. C.S. Johnson, Diffusion ordered nuclear magnetic resonance spectroscopy: principles and applications, *Progress in Nuclear Magnetic Resonance Spectroscopy,* **34**, 203-256 (1999).
53. C.R. Cantor, and P.R. Schimmel, *Biophysical Chemistry, Part II: Techniques for the Study of Biological Structure and Function,* W.H. Freeman, New York, NY, pp. 539–590, (1980).
54. J.Cavanagh, W. J. Fairbrother, A. G. Palmer III, and N. J. Skelton, *Protein NMR Spectroscopy. Principles and Practice*, Academic Press, Inc (1996).
55. I. Bertini, and C. Luchinat, *Coord. Chem. Rev.* **150**, 1-296 (1996).
56. F. A. Cotton, G. Wilkinson, *Advanced Inorganic Chemistry, a comprehensive text*, John Wiley and Sons, Inc., New York (1980).
57. H.Y. Shrivastava, M. Kanthimathi, and B.U. Nair, *Biochim. Biophys. Acta* **1573**, 149-155 (2002).
58. Hypercube, Hyperchem, release 5.0. Reference manual. Waterloo, Ontario., CN: Hypercube Inc., 1997.

